# Interaction of Oxidative Stress and Misfolded Proteins in the Mechanism of Neurodegeneration

**DOI:** 10.3390/life10070101

**Published:** 2020-06-30

**Authors:** Andrey Y. Abramov, Elena V. Potapova, Viktor V. Dremin, Andrey V. Dunaev

**Affiliations:** 1 Department of Clinical and Movement Neurosciences, UCL Queen Square Institute of Neurology, Queen Square, London WC1N 3BG, UK; 2 Cell Physiology and Pathology Laboratory, Orel State University, 302026 Orel, Russia; e.potapova@oreluniver.ru (E.V.P.); v.dremin@oreluniver.ru (V.V.D.); dunaev@bmecenter.ru (A.V.D.); 3 Aston Institute of Photonic Technologies, School of Engineering and Applied Science, Aston University, Birmingham B4 7ET, UK

**Keywords:** neurodegeneration, reactive oxygen species, β-amyloid, α-synuclein, tau protein, mutant huntingtin protein, oxidative stress

## Abstract

Aggregation of the misfolded proteins β-amyloid, tau, huntingtin, and α-synuclein is one of the most important steps in the pathology underlying a wide spectrum of neurodegenerative disorders, including the two most common ones—Alzheimer’s and Parkinson’s disease. Activity and toxicity of these proteins depends on the stage and form of aggregates. Excessive production of free radicals, including reactive oxygen species which lead to oxidative stress, is proven to be involved in the mechanism of pathology in most of neurodegenerative disorders. Both reactive oxygen species and misfolded proteins play a physiological role in the brain, and only deregulation in redox state and aggregation of the proteins leads to pathology. Here, we review the role of misfolded proteins in the activation of ROS production from various sources in neurons and glia. We discuss if free radicals can influence structural changes of the key toxic intermediates and describe the putative mechanisms by which oxidative stress and oligomers may cause neuronal death.

## 1. Introduction

With an increase in life expectancy, the prevalence of age-related neurodegenerative diseases has dramatically increased. Neurodegenerative disorders are associated with neuronal loss in specific brain regions. This leads to a progressive decline in cognitive function (such as Alzheimer’s disease or frontotemporal dementia) and/or movement (Parkinson’s disease, amyotrophic lateral sclerosis (ALS)). Neurodegenerative disorders are mostly regarded as a sporadic disease and with genetic factors also playing a role in disease pathogenesis. Importantly, the main characteristics for both sporadic and familial forms of neurodegenerative disorders are the deposition and spreading of aggregated proteins, oxidative stress, chronic neuroinflammation, and mitochondrial dysfunction, causing neuronal loss [1].

Oxidative stress is the oxidative damage of biological molecules which leads to abnormal function and initiation of cell death. It is induced by an imbalance between the production of free radicals (including reactive oxygen species (ROS)) and the efficiency of the antioxidant system [2]. ROS are produced in the cells enzymatically (mitochondrial enzymes, NADPH oxidase, xanthine oxidase, etc.) and non-enzymatically (UV) and play multiple physiological roles in the brain cells [3]. The reactivity and toxicity of oxygen depends on its electronic structure. Kinetically stable oxygen contains the identical spin states of its two outer orbital electrons. Partially reduced forms of oxygen which contain a free radical are very unstable and must either accept or be a donor of electrons. Considering this, they are very active and have a very short lifetime. There are various partially reduced ROS including oxygen radicals: superoxide (O_2_•−) and the hydroxyl radical (OH•). Although hydrogen peroxide (H_2_O_2_), singlet oxygen (^1^O_2_), or ozone (O_3_) are non-radicals, they can be easily converted to free radicals and also named “ROS”. High reactive activity of ROS and ability for fast chemical interaction with biological molecules is adopted by cells for fast signaling or regulatory purposes, which is controlled by a highly effective antioxidant system.

Overproduction of ROS or/and deregulation of the antioxidant system can lead to oxidation of proteins, DNA, or lipid peroxidation that severely alter cell homeostasis.

Aggregated misfolded proteins are specific for each neurodegenerative disease: β-amyloid, which forms extracellular senile plugs, and aggregates of tau protein (major component of intracellular tangles) are the histopathological features of Alzheimer’s disease [4]. Parkinson’s disease is associated with aggregated α-synuclein in intracellular occlusions called Lewy bodies in the brain stem, neocortical regions, and spinal cord [5].

It should be noted that some of the aggregated proteins are not linked only to a specific disease. Thus, α-synuclein aggregation (synucleinopathy) is also characterized as dementia with Lewy bodies and multiple system atrophy [6], while tau aggregates are not specific for Alzheimer’s only and appear in a number of neurodegenerative disorders, tauopathies, including primary age-related tauopathy, progressive supranuclear palsy (PSP), frontotemporal dementia, and parkinsonism linked to chromosome 17, Pick’s disease, and corticobasal degeneration [7]. Importantly, mutation in the alpha-synuclein gene (SNCA) leads to Parkinson’s disease, and the Tau gene FTDP-17 with 10 + 16 MAPT mutation is shown to be linked to a familial form of frontotemporal dementia [8]. Familial forms of Alzheimer’s disease are also associated with mutations in the production of β-amyloid [9], confirming the importance of misfolded proteins in the development of pathology in neurodegenerative diseases. One of the main histopathological features of Huntington’s disease is the aggregation of huntingtin protein [10,11]. All these aggregates in the brain consist mostly of protein fibrils, which are predominantly non-toxic, and more likely toxic small oligomeric forms of these proteins are the trigger of cellular pathology and neurodegeneration in these diseases. Misfolded proteins could be seeded and spread in neurons and some astrocytes in a prion-like mechanism [12].

Most of these proteins play a physiological role in monomeric form. Thus, α-synuclein is shown to be important in synaptic transmission and mitochondrial bioenergetics [13,14,15]. Tau is a microtubule-associated protein which also plays a role in stabilizing neuronal microtubules and thus promotes axonal outgrowth [16]. Transformation to a toxic form for these proteins requires aggregation, and for tau, phosphorylation is also needed. This is distinctive and a multifactual process which may be dependent on the oxidative state of the cells, and an aggregated form of β-amyloid or α-synuclein can be formed with metal-ions such as copper, iron, or zinc [17]. Transition metals can produce ROS, and considering this, aggregated misfolded proteins require ROS for aggregation but can also be the source of ROS production [18].

One of the major problems associated with neurodegenerative disorders is finding the biomarkers for early diagnostics, and, importantly, both aggregated misfolded proteins and products of oxidative stress are currently tested as potential biomarkers for neurodegenerative diseases.

In this review, we summarize the role of ROS and the products of oxidation in the mechanism of pathology and misfolding and the accumulation of abnormally aggregated proteins.

## 2. α-synuclein and Oxidative Stress

The most common neurodegenerative movement disorder is also the second most common case of dementia. Lewy bodies and aggregated α-synuclein are essential histopathological hallmarks of degenerating neurons in the brains of patients with Parkinson’s disease, but also for a group of neurodegenerative disorders, a variety of clinical syndromes underlies, including movement disorders/parkinsonism (Parkinson’s disease, pantothenate kinase-associated neurodegeneration), dementias (dementia with Lewy body and Parkinson’s disease dementia), and autonomic dysfunction (pure autonomic failure, multiple system atrophy).

The role of oxidative stress in the pathology of Parkinson’s disease was established in toxic (MPP+) models and cells from sporadic Parkinson’s patients [19,20].

There is a growing number of publications demonstrating the importance of synucleins and, particularly, α-synuclein in physiology. In fact, the physiological form of α-synuclein is non-toxic and does not activate ROS production in neurons and glia [21,22]. The monomeric form of α-synuclein is a soluble protein which aggregates to a structure which forms insoluble Lewy body fibrils via several conformational changes including the most toxic oligomeric intermediates.

**Figure 1:**
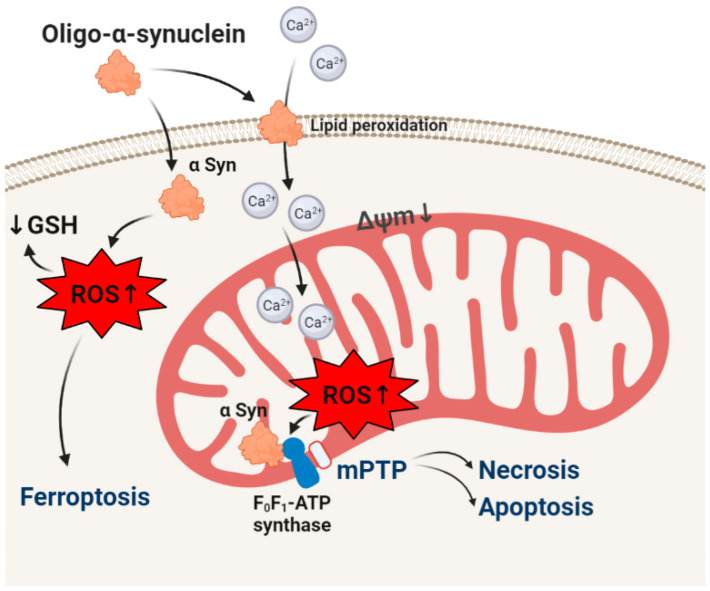
Effect of oligomeric α-synuclein on the redox balance in neurons. α-Synuclein-induced reactive oxygen species (ROS) lead to decreased endogenous antioxidant (GSH), lipid peroxidation, and oxidation. Although oligomeric α-synuclein has no effect on ROS production in mitochondria, α-synuclein produced ROS oxidase mitochondrial proteins including subunits of F_1_-F_0_-ATPase. In combination with calcium overload, it induces mitochondrial permeability transition pore (mPTP) opening and cell death.

Although α-synuclein is an intracellular protein, all forms (monomeric, oligomeric, and fibrillar) are able to pass through plasma and intracellular membranes [21,23]. Importantly, application of different forms of α-synuclein to primary neurons or human neurons induced ROS production and a decrease in the level of endogenous antioxidant (GSH) only when the toxic oligomeric form was present [21,22]. Oligomer-induced ROS production and oxidative stress were shown to be independent of inhibitors of NADPH oxidase and xanthine oxidase, and importantly, monomers, oligomers, and fibrils had no effect on the rate of ROS production in mitochondria that suggests a non-enzymatic way of ROS production [22,24,25]. Oligomers, in contrast to monomers and fibrils, can produce ROS in vitro by themselves (Figure 1) [26]. Importantly, α-synuclein (oligomer)-induced ROS production in salt solution or in cells can be blocked by chelators of copper, iron, or zinc [22,26]. This ROS production is less likely to be produced only by transition metals in the medium, because the same medium was used for monomers and fibrils which did not produce ROS and is possibly connected to some structural changes in the α-synuclein molecule induced by these metals ions, although α-synuclein binds these metals in very small amounts [27,28,29].

This ability of oligomeric α-synuclein to generate ROS may be important in the mechanism of neurotoxicity. Thus, α-synuclein can form ion channels and initiate calcium signal in neurons and astrocytes (Figure 1) [30,31]. Interaction of oligomers with lipids leads to lipid peroxidation [32], and oxidized lipids increase the α-synuclein-induced channel formation and calcium signal [33].

Although α-synuclein has no effect of ROS production in mitochondria, ROS produced by oligomeric α-synuclein target mitochondrial function. Physiological monomeric α-synuclein binds to F_0_-F_1_-ATPsythase and increases efficiency in ATP production [14]. Oligomeric α-synuclein binds to the same subunit of this mitochondrial enzyme. Most of ROS toxicity, due to their short lifetime, is mainly limited to the site of their origination. Production of superoxide by oligomer oxidizes proteins of F_0_-F_1_-ATPsynthase and in combination with mitochondrial calcium overload leads to forming and opening of the mitochondrial permeability transition pore and consequently to cell death [26]. It should be noted that oligomeric α-synuclein is involved in triggering apoptosis [32], necrosis [24,26], and ferroptosis [33].

Although acute action of α-synuclein did not induce any effect on enzymatic ROS production in neurons and astrocytes, in human neurons with α-synuclein triplication, part of the ROS overproduction was sensitive to inhibitors of NADPH oxidase [22]. Importantly, oligomeric α-synuclein or oligomers with A53T and A30P mutations are shown to be activators of NADPH oxidase in microglia that trigger inflammation [34,35]. In agreement with that, it was shown that inhibiton of NADPH oxidase apocynin prevents learning and memory deficits in a mouse Parkinson’s disease model [36]. Selective activation of NADPH oxidase by α-synuclein in microglia compare to other cell types could be potentially explained by direct integration of extracellular oligomers with the microglial P2X7 receptor [37].

Oligomeric α-synuclein binds with chaperons that change activity of this protein including its ability to produce ROS [38] that can be neuroprotective. However, this ability may have a negative effect on cell function as well. Thus, oligomeric α-synuclein interacts with the Hsp70 system that inhibits the chaperone activity by weak interactions with J-domain co-chaperones that may contribute to the disruption of protein homeostasis, impair organellar function, and contribute to the mechanism of neurodegeneration in Parkinson’s disease [39], but on the other hand, it can be a part of a natural neuronal control strategy to suppress α-synuclein aggregation [40].

Such an intensive α-synuclein-induced production of ROS in the cells lead to oxidation of DNA and activation of DNA repairing enzymes. Thus, ROS activates poly (ADP-ribose) polymerase (PARP) activity in Parkinson’s disease-related Fbxo7-deficient cells that induce alteration of the energy metabolism via depletion of the NAD+ content [41]. Oligomeric and mutated α-synuclein activates PARP-1, generating PAR that accelerates the further formation of pathologic α-synuclein, resulting in cell death via parthanatos (poly ADP ribose polymerase-mediated cell death). Importantly, a high level of PAR is shown in cerebrospinal fluid of patients with Parkinson’s disease [42].

## 3. Tau, Tauopathies, and Oxidative Stress

Tauopathies, including progressive supranuclear palsy, corticobasal syndrome, most frontotemporal dementias, chronic traumatic encephalopathy, and importantly, Alzheimer’s disease, are progressive neurodegenerative disorders with tau deposits as a histopathological feature in the brain. Although intracellular tangles were known in Alzheimer’s for decades, only in 1975, a protein contaminant that co-purified with microtubules was identified which was later associated with pathological tangles and named tau [43,44].

Tau is a microtubule-associated protein (MAP) that is encoded by the MAPT gene. This protein is known to interact with α- and β-tubulin in aid of microtubule assembly. With age, tau enriches and aggregates in axons and dendrites. Tau can exist in six isoforms generated through alternative mRNA splicing and can be phosphorylated at multiple possible sites [45]. Phosphorylation may lead to oligomerization, and this transition from an intrinsically disordered monomer to a highly structured filament is recognized to drive pathogenesis in tauopathies [46]. Tau aggregates exhibit cell–cell transfer that leads to seeding and further aggregation, which underpins the region–region spread of disease in tauopathies [46].

**Figure 2:**
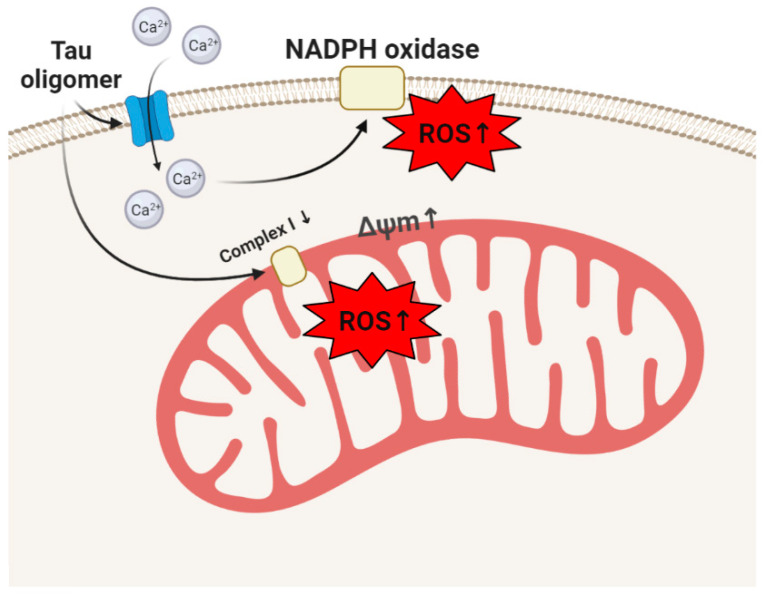
Phosphorylated tau forms a channel on the neuronal membrane and induces a calcium signal which activates ROS production in NADPH oxidase. Tau aggregates inhibit mitochondrial respiration and induce ROS overproduction in this organelle.

Application of extracellular tau at different stages of aggregation to cortical co-cultures of neurons and astrocytes showed that only insoluble aggregates of tau are able to induce ROS production by activation of NADPH oxidase via a calcium-dependent way (Figure 2) [47]. However, it was not associated with a decrease in the level of GSH and could be associated with physiological processes. Possibly, it can activate tau production because antioxidants and NADPH-oxidase (NOX) inhibitors are shown to effectively reduce the expression of tau and MAP2 [48]. Activation of NADPH oxidase in combination with membrane-active properties of tau aggregates induced neuronal cell death [47].

Tau protein effectively induces ROS production in mitochondria. Thus, in human iPSC-derived neurons with the 10+16 intronic mutation in MAPT gene (FTDP-17) encoding tau, mild inhibition of mitochondrial complex I switches the F_1_F_0_ ATPase to reverse mode for maintenance of the mitochondrial membrane potential [49]. This type of maintenance of mitochondrial potential in cells with complex I inhibition may lead to mitochondrial hyperpolarization which induces excessive ROS production (Figure 2) [50]. This mechanism of ROS overproduction in mitochondria in neurons with FTDP-17 triggered cell death which could be prevented by incubation of these cells with mitochondrial antioxidants [49]. Importantly, these neurons are associated with altered electrophysiology which can also be damaged by oxidative stress [51]. Similar mechanisms of oxidative stress via mitochondrial ROS overproduction were found in mesenchymal stem cells (MSCs) derived from the bone marrow of patients with another form of tauopathy–progressive supranuclear palsy [52]. Hippocampal phosphorylated tau in tau mice with P301L mutation also induced mitochondrial disfunction resulting in ROS (hydrogen peroxide) production and lipid peroxidation, which was shown to be the trigger for neuronal loss [53,54].

Potentially, mitochondrial antioxidants could have a positive effect in these forms of tauopathies, although normalization of the mitochondrial metabolism and ROS production by Nrf2 activation can also be effective as a therapeutic strategy for these diseases [55,56].

Various antioxidants are shown to be effective in cellular and mouse models of Alzheimer’s disease, including hyperphosphorylated tau models. However, it is not clear if these antioxidants reduce the oxidative damage produced by tau or decrease the ROS-reduced hyperphosphorylation of tau in these models of tauopathy [57].

## 4. β-amyloid and Oxidative Stress in the Mechanism of Neurodegeneration

The most common neurodegenerative disorder was described more than 100 years ago by Alois Alzheimer but still remains uncurable. Alzheimer’s disease is the most common case for dementia in older people. Although symptoms of sporadic Alzheimer’s disease start to appear after 60 years of age, the actual neurodegenerations occur years earlier. Oxidative stress has been proven to be one of the major triggers for Alzheimer’s disease pathology for decades. Thus, products of lipid peroxidation were shown to be elevated in blood samples and brain autopsy of patients of Alzheimer’s disease [58,59]. β-amyloid is a main component of the senile plaques which are surrounded by activated microglia which could already be proof of involvement of this peptide in the initiation of the ROS production. β-amyloid is produced from the amyloid precursor protein by a cleavage by β- and γ-secretase generating a peptide ranged between 39 and 43 amino acid residues long, where the hydrophobic nature of β-amyloid 1-40 and 1-42 promotes self-aggregation and neurotoxicity.

There is a link between β-amyloid and redox metal dysregulation, which was supported by post-mortem analysis of amyloid plaques, which revealed accumulation of copper, iron, and zinc [60]. Considering the involvement of redox metals in the accumulation of β-amyloid, the generation of ROS by complex β-amyloid-transition metal was suggested [61]. It can be partially confirmed by the effects of heavy metal chelators on the β-amyloid-induced oxidative stress; however, it can also be explained by the structural changes of β-amyloid in the absence of metal ions. The second hypothesis is more probable because production of ROS by β-amyloid by itself in vitro was not shown [62].

**Figure 3:**
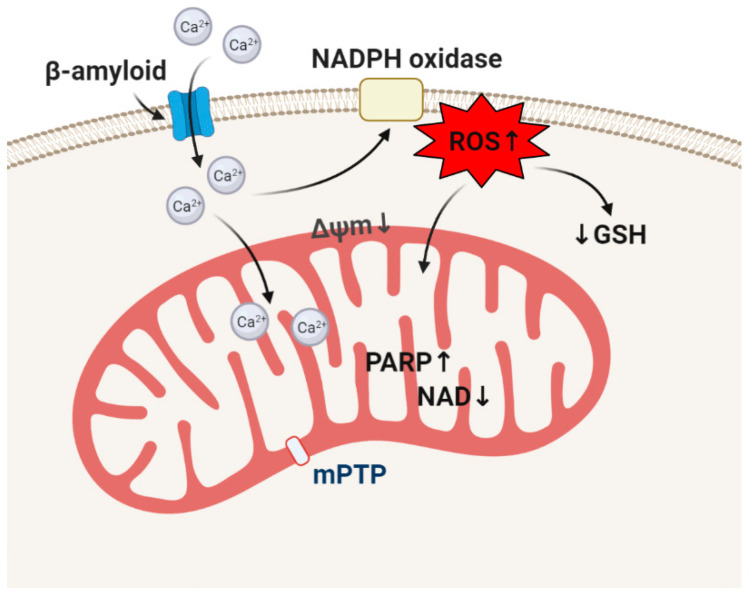
Effect of β-amyloid on redox homeostasis and mitochondrial metabolism of astrocytes.

β-amyloid more effectively incorporates in the membranes of astrocytes and forms pores which can stimulate a calcium signal because of a higher cholesterol level in these cells compared to neurons (Figure 3) [63,64]. A β-amyloid-induced calcium signal in astrocytes activates NADPH oxidase in these cells that induces GSH decrease, activation of DNA-repairing enzyme poly(ADP-ribose) polymerase, mitochondrial depolarization, and neuronal cell death (Figure 3) [65,66,67]. Importantly, β-amyloid is neurotoxic and is able to stimulate a calcium signal and activate NADPH oxidase only in oligomeric but not in monomeric or fibrillar form. Oligomers can be active even in picomolar concentrations [68]. The hormone melatonin, which is decreased with age, also possesses antioxidant properties. Melatonin can suppress the action of β-amyloid on the ROS production and neurotoxicity [69,70].

β-Amyloid-induced NADPH oxidase activation in combination with generation of nitric oxide can stimulate peroxinitrite generation which is a trigger for neurotoxicity [71,72].

However, the effect of β-amyloid on ROS production and the level of endogenous antioxidants depend on the aggregation and on the cell type—it predominantly activates ROS production in microglia and astrocytes that induce oxidative stress and cell death in neurons.

## 5. Huntingtin and Oxidative Damage

Huntington’s disease is a devastating neurodegenerative disorder characterized by chorea motor impairment and gradual intellectual decline which lead to psychiatric illness, pathologically by loss of long projection neurons in the cortex and striatum [73]. This disease is associated in with mutations in the huntingtin protein (Htt) characterized by multiple CAG (Gln) repeats. The expansion of polyglutamine repeats leads to altered Htt conformation, which activates aggregation of this protein. As a result of this specific mutation, the protein huntingtin is modified—mutant huntingtin protein (mHtt). The mHtt or its fragments are capable of initiating a damage cascade of molecular processes, which ultimately results in mitochondrial dysfunction, formation of ROS, and elevated oxidative stress [74,75,76,77,78,79]. Oxidative damage has been confirmed by immunohistochemical data and in biochemical tests in patients with Huntington’s disease [80,81,82].

Intracellular protein aggregation directly causes free radical production and subsequent cellular damage, with ROS production dependent on the amount of glutamine residues [83]. Mutant huntingtin causes aberrant transcription regulation by binding to several transcription regulators and disrupting their function. Mitochondrial disorders in Huntington’s disease can go by reducing the expression of the main metabolic co-regulator, which plays an important role in suppressing oxidative stress–PGC-1α [84]. Several mechanisms of supposed PGC-1α-mediated stress are described. Cui et al. demonstrated that mHtt prevents the CREB/TAF4 complex formation that regulates the transcription and expression of the PGC-1α gene [85].

mHtt induces the production of ROS mainly in mitochondria by direct interaction with mitochondrial proteins, as translocase of the inner membrane 23, disrupting mitochondrial proteostasis and inducing ROS production; considering this, mitochondrial and redox-based therapeutic strategies are intensively discussed for Huntington’s disease [86].

Another major producer of ROS in Huntington’s disease is NADPH oxidase. Thus, polyQ-expanded proteins, including native huntingtin, are interact with gp91, the major membrane NADPH-oxidase -2 subunit [87].

One important function of the p53 protein is its involvement in antioxidant protection through activation of a group of genes, encoding a set of antioxidant proteins [88]. The pathogenic domain of mutant Htt interacts with critical cellular transcription factors and potentially modulates p53-induced transcriptional events in cells [89].

Another possible way to influence mHtt by oxidative stress is to disrupt the expression of cystathionine γ-lyase (CSE) by sequestration of SP1, the main transcription factor for CSE [90,91]. Low levels of CSE and cysteine transporters lead to decreased levels of cysteine in cells, leading to increased oxidative stress.

Another evidence that huntingtin may induce oxidative stress not through overproduction of ROS but by decrease in the level of antioxidant defense is shown for full-length mutant Htt (FL-mHtt). Thus, cells with expression of FL-mHtt are shown to have decreased glutamate-cysteine ligase and glutathione synthetase activities suggesting decreased de novo synthesis of glutathione. In combination with inhibition of activity of the multidrug resistance protein 1 which mediates cellular export of glutathione conjugates, it maintains GSH-related antioxidant defense mechanisms insufficiently to protect cells against basic ROS production [92].

Another possible way of participation of mHtt in the development of oxidative stress is the decrease in expression of a transcription factor, the nuclear factor -κB (NF-κB), which mediates antioxidant and antiapoptotic signaling as a response of the endoplasmic reticulum to a stress [93].

It has been reported that mHtt fragments bind to the translocase of the inner mitochondrial membrane 23 (TIM23) complex and inhibit the mitochondrial protein import by altering the mitochondrial proteome, leading to mitochondrial dysfunction [94,95].

Mutant huntingtin significantly decreases the Ca^2+^ threshold and directly induces the opening of the mitochondrial permeability transition pore (mPTP) [96,97,98].

The balance of mitochondrial fission and fusion is significantly impaired in Huntington’s disease, leading to accumulation of fragmented and damaged mitochondria followed by increased oxidative stress [99]. It is believed that the disruption of the mitochondrial fission–fusion balance and mitochondrial transport along axons and dendrites occurs through direct interaction of mHtt with Drp1 [100,101] or through modulation of S-nitrosylation of Drp1 [102].

## 6. Conclusions

There is a growing number of publications reporting evidence of the involvement of misfolded proteins in the mechanism of oxidative damage in neurodegenerative disorders. Despite the fact that most of the aggregated proteins have a strong effect of oxidative stress, the mechanism of ROS production or the effect on the antioxidant system is different. Thus, the most active among the misfolded proteins is the β-amyloid which activates ROS production in microglia and astrocytes via the activation of NADPH oxidase; oligomeric α-synuclein can generate ROS by itself, while tau and huntingtin activate the production of ROS in the mitochondrial electron transport chain. This excessive ROS production targets various pathways that lead to differences in the types of neurodegeneration.

Although implication of the aggregated misfolded proteins in ROS production and oxidative stress in neurodegeneration is clearly stated by a number of publications on different levels—from single molecules to in vivo models and patient post brain samples—all clinical trials in neurodegenerative disorders based on antioxidant therapy were unsuccessful. This phenomenon is typical not only for neurodegenerative disorders and related to many factors, including antioxidant delivery, quenching of the antioxidant properties before it reaches the oxidized area, etc. Considering this, it is difficult to generalize an antioxidant strategy for these disorders, and therefore, each neurodegenerative disease demands a highly targeted treatment. Non-antioxidant, targeted protection against oxidative stress, including transition metal chelators, compounds which modify oligomeric structure and inhibitors of enzymatic ROS production (such a NADPH oxidase) may potentially have a strong therapeutic effect against neurodegenerative disorders, and these directions are now rapidly developing. Although misfolded proteins activate ROS production and induce oxidative stress, these processes cannot be separated from other mechanisms which occur in the brain during the development of pathology.
